# Complete Genome Sequence of Citrobacter freundii Myophage Maroon

**DOI:** 10.1128/MRA.01145-19

**Published:** 2019-10-24

**Authors:** James R. McDermott, Qiuyan Shao, Chandler O’Leary, Rohit Kongari, Mei Liu

**Affiliations:** a Center for Phage Technology, Texas A&M University, College Station, Texas, USA; Loyola University Chicago

## Abstract

Citrobacter freundii is a nosocomial opportunistic pathogen that can cause urinary and bloodstream infections. Phage therapies against C. freundii may prove useful in treating infections caused by this ubiquitous bacterium. Here, we report the complete genome of a T4-like myophage, Maroon, that infects C. freundii.

## ANNOUNCEMENT

Citrobacter freundii is a facultative anaerobic Gram-negative bacterium that can cause opportunistic nosocomial infections within the bloodstream, respiratory tract, and urinary tract in immunocompromised patients (1, 2). With the increase of multidrug-resistant *Citrobacter* infections (3), phage therapy is being investigated as an alternative solution (4, 5). Here, we present the complete genome of the C. freundii myophage Maroon.

Phage Maroon was isolated from a municipal wastewater sample collected from Brazos County, Texas, in 2015 using a C. freundii strain as the host. LB broth or agar (Difco) was used to culture the host bacterium and phage enrichment at 37°C with aeration. Phage isolation and propagation were conducted using the soft-agar overlay method (6). It was identified as a siphophage using negative-stain transmission electron microscopy performed at the Texas A&M University Microscopy and Imaging Center as described previously (7). Phage genomic DNA was prepared using a modified Promega Wizard DNA cleanup kit protocol (7). Pooled indexed DNA libraries were prepared using the Illumina TruSeq Nano LT kit, and the sequence was obtained from the Illumina MiSeq platform using the MiSeq V2 500-cycle reagent kit following the manufacturer’s instructions, producing 616,387 paired-end reads for the index containing the Maroon genome. FastQC 0.11.5 (https://www.bioinformatics.babraham.ac.uk/projects/fastqc/) was used to quality control the reads. The reads were trimmed with FastX Toolkit 0.0.14 (http://hannonlab.cshl.edu/fastx_toolkit/download.html) before being assembled using SPAdes 3.5.0 (8). Contig completion was confirmed by PCR using primers (5′-CCTGGGATATCCGTAATTGG-3′ and 5′-TATCGAAGCCATTTTGACCA-3′) facing off the ends of the assembled contig and Sanger sequencing of the resulting product, with the contig sequence manually corrected to match the resulting Sanger sequencing read. GLIMMER 3.0 (9) and MetaGeneAnnotator 1.0 (10) were used to predict protein-coding genes with manual verification, and tRNA genes were predicted with ARAGORN 2.36 (11). Rho-independent termination sites were identified using Transterm (http://transterm.cbcb.umd.edu/). Sequence similarity searches were done using BLASTp 2.2.28 (12) against the NCBI nonredundant (nr), UniProt Swiss-Prot (13), and TrEMBL databases with a 0.001 maximum expectation value cutoff. InterProScan 5.15-54.0 (14), LipoP (15), and TMHMM 2.0 (16) were used to predict protein function. All analyses were conducted at default settings via the CPT Galaxy (17) and Web Apollo (18) interfaces (https://cpt.tamu.edu/galaxy-pub).

Maroon was assembled at 32.2-fold coverage into a 178,830-bp genome. The GC content of the genome is 44.9%. A total of 277 coding sequences were annotated, resulting in a protein-coding density of 95%. Maroon shares nucleotide-level similarity across the genome with many T4-like phages, such as *Citrobacter* phage Margaery (GenBank accession no. KT381880) and *Cronobacter* phage vB_CsaM_leB (GenBank accession no. KX431559) (sharing 99% and 94% DNA similarity, respectively). While 168 of the identified genes do not have a predicted function, proteins with annotated functions have homologs (via BLASTp against the NCBI nr database at an E value of <0.001) in T4 (GenBank accession no. NC_000866) or T4-like phages. Genes involved in DNA replication and nucleotide biosynthesis (DNA helicase, *nrdA*, *nrdB*, polynucleotide kinase, etc.) and virion morphogenesis (capsid proteins, tail fibers, tail sheath stabilizer, tail baseplate, etc.) were all identified. Lysis genes coding for a class III holin, a soluble l-alanyl-d-glutamate peptidase, and a separated spanin pair were also found.

### Data availability.

The genome sequence of phage Maroon was deposited under GenBank accession no. MH823906. The associated BioProject, SRA, and BioSample accession numbers are PRJNA222858, SRR8770019, and SAMN11233125, respectively.
